# Rapid Weight Regain and Methods of Victory Among Winning Professional Mixed Martial Artists

**DOI:** 10.7759/cureus.104442

**Published:** 2026-02-28

**Authors:** Corey Peacock, Peter Byers, Arjun Mote, Jose Antonio, Gabriel J Sanders

**Affiliations:** 1 Department of Health and Human Performance, Nova Southeastern University Dr. Kiran C. Patel College of Osteopathic Medicine, Davie, USA; 2 Exercise Physiology, Kent State University, Kent, USA; 3 Department of Biological Sciences, Nova Southeastern University Halmos College of Arts and Sciences, Davie, USA; 4 Exercise Science, University of Cincinnati, Cincinnati, USA

**Keywords:** mma, professional athletes, rapid weight regain, sports performance, victory

## Abstract

Introduction: Rapid weight loss and subsequent rapid weight regain (RWR) are common practices in professional mixed martial arts (MMA), driven by weight-category regulations and competitive strategy. Although RWR is often assumed to confer a performance advantage, limited research has examined whether the magnitude of weight regained influences how bouts are won among successful fighters.

Methods: This observational study analyzed publicly available regulatory records from Ultimate Fighting Championship and Bellator MMA events sanctioned by the California State Athletic Commission between 2018 and 2024. Only winning fighters were included. Official weigh-in and fight-night body mass were recorded using commission-calibrated scales. RWR was calculated as the percentage change from official weigh-in to fight-night weight. Independent samples t-tests were used to compare RWR by organization and sex division, and one-way analysis of variance was used to examine differences by method of victory. Statistical significance was set at p≤0.05.

Results: A total of 154 winning professional MMA fighters were included. Mean RWR was 10.17±3.66%. RWR did not differ significantly between organizations (p=0.147) or between sex divisions (p=0.354). Method of victory was not impacted by RWR, with no significant differences observed between knockout or technical knockout, submission, or decision outcomes (p=0.383).

Conclusion: RWR is prevalent among winning professional MMA fighters; however, the magnitude of post-weigh-in weight regain does not appear to influence the method of victory. These findings suggest that while weight regain is a common component of fight preparation, it may not be a determining factor in how competitive success is achieved.

## Introduction

Mixed martial arts (MMA) is a full-contact combat sport that combines a wide range of fighting techniques, including boxing, Brazilian jiu-jitsu, wrestling, Muay Thai, and judo [1,2]. Since its early, largely unregulated origins, MMA has evolved into a globally recognized sport governed by athletic commissions, with standardized rules, weight divisions, and safety regulations that have contributed to its mainstream acceptance [1]. The Ultimate Fighting Championship (UFC), founded in 1993, has played a central role in this evolution and is now the dominant professional MMA organization worldwide, valued at over $11 billion [3,4]. In addition to the UFC, other prominent organizations such as Bellator MMA and ONE Championship have contributed to the sport’s global expansion [5].

Modern MMA competitions follow the Unified Rules of MMA, which were first adopted in 2000 and outline prohibited techniques, bout structure, and judging criteria [6]. Non-title bouts typically consist of three five-minute rounds, while championship bouts and main events consist of five rounds. Victories are achieved through knockout (KO) or technical knockout (TKO), submission, or judges’ decision. To promote competitive equity, fighters compete within designated weight categories, and official weigh-ins are conducted approximately one day prior to competition. Failure to make the contracted weight may result in fines, bout cancellation, or catchweight agreements. As a result, weight management has become an integral component of fight preparation.

Despite increased regulation, the widespread practice of rapid weight loss (RWL) remains one of the most controversial aspects of MMA. Fighters routinely engage in aggressive weight-cutting strategies to qualify for a specific weight category, often involving dehydration, caloric restriction, sauna use, water loading, and electrolyte manipulation [7-9]. Accordingly, RWL practices are reported in approximately 60% of athletes in judo, jiu-jitsu, karate, and taekwondo and in over 90% of MMA athletes, with evidence indicating that at official weigh-ins, 57% of MMA competitors are dehydrated and 43% are severely dehydrated [7,10-12]. These practices can result in acute reductions of 10%-15% of body mass within a short time frame [11]. Prior investigations in UFC athletes have reported an average body mass loss of approximately 6%-8% in the 72 hours preceding weigh-ins, with notable variability across weight categories and sex divisions [12,13].

RWL is part of MMA and has been associated with numerous physiological and health-related consequences, including severe dehydration, electrolyte imbalance, reduced plasma volume, cardiovascular strain, renal stress, hormonal disruption, and impaired cognitive and physical performance [14-16]. In extreme cases, improper weight-cutting practices have resulted in collapse or death, drawing increased scrutiny from medical professionals and regulatory bodies [17]. Despite these risks, RWL persists within MMA culture, largely due to the perception that failing to cut weight places athletes at a competitive disadvantage [10].

Following official weigh-ins, fighters typically engage in rapid weight regain (RWR) during the limited recovery window prior to competition. After exercise-induced sweat loss and RWL, rehydration and nutritional refeeding strategies aim to restore body mass, plasma volume, glycogen stores, and overall physiological function [18,19]. Previous research has demonstrated that MMA fighters often regain more than 10% of their body mass within 24 hours of weigh-ins [10,11]. In UFC athletes, the mean post-weigh-in weight regain of approximately 9% to 10% has been reported, which theoretically supports the premise that restoring body mass after acute weight reduction may provide a size-related and potentially competitive advantage over opponents in certain combat sport contexts [12]. However, despite the prevalence of RWR, concerns remain regarding incomplete fluid replacement and its effects, including impaired physiological recovery, persistent dehydration, and residual performance impairments at the time of competition [15,20].

While RWR is commonly believed to confer a competitive advantage, the relationship between RWR and competitive success remains unclear. Prior investigations in both UFC and Bellator MMA athletes have reported no significant association between the magnitude of post-weigh-in weight regain and fight outcomes [10,21,22]. Importantly, limited research has examined whether RWR influences the method by which victories are achieved among winning fighters. Understanding whether RWR differentially impacts KO, submission, or decision outcomes may provide important insight into the practical relevance of weight-cutting practices beyond general win-loss outcomes.

A portion of this work was previously presented as a meeting abstract at the 22nd Annual ISSN Conference on June 24, 2025; however, a larger dataset was used for the present analysis. Therefore, the purpose of this study was to examine RWR among winning professional MMA fighters competing in UFC and Bellator MMA events sanctioned by the California State Athletic Commission (CSAC), and to determine whether the magnitude of weight regain differed by organization, sex division, or method of victory. Based on prior literature, it was hypothesized that RWR would not significantly impact the method of victory among winning fighters.

## Materials and methods

This study was an observational analysis of publicly available regulatory records. Professional MMA fighters who competed in sanctioned UFC and Bellator MMA events in California, USA, between 2018 and 2024 were included. Data were obtained from publicly released official records provided by the CSAC. A total of 13 CSAC-sanctioned events, held across multiple cities in California, were included. All events were overseen by the same athletic commission and followed identical regulatory and weigh-in procedures. Only winning fighters from these events were included in the present analysis, resulting in a final sample of 154 professional athletes. This approach was used to focus on weight cutting and regain practices in the context of successful competitive outcomes, building on preliminary findings previously presented. Fighters who lost their bouts were not included in this dataset.

All body mass measurements were conducted using CSAC-calibrated scales and were supervised by CSAC officials. The official weigh-in occurred between 9:00 am and 11:00 am, approximately 36 hours prior to the competition, in accordance with standard CSAC regulations. Fight-night body mass was measured upon the athlete's arrival at the competition venue on the day of the event. These weigh-in procedures were mandatory for all fighters as part of CSAC event requirements. RWR was calculated as the percentage change in body mass from the official weigh-in to fight-night weight. This measure reflects acute post-weigh-in mass regain following RWL strategies commonly used to meet official weight limits.

All data analyzed in this study were publicly available, deidentified, and derived from official regulatory records. Fighter participation in weigh-in procedures was mandatory under CSAC event rules. The study protocol (IRB #2024-355) was reviewed by the Institutional Review Board (IRB) and determined to be outside the purview of IRB oversight, as it did not involve human subjects. Therefore, IRB approval and informed consent were not required.

Descriptive statistics are reported as mean±standard deviation. Independent samples t-tests were used to examine differences in RWR between organizations (UFC vs. Bellator MMA) and between sex divisions (women’s divisions vs. men’s divisions). Differences in RWR by method of victory (KO/TKO, submission, and decision) were examined using a one-way analysis of variance. Statistical significance was set a priori at p≤0.05. All analyses were performed using standard statistical software (SPSS 27, Armonk, NY).

## Results

A total of 154 winning professional MMA fighters were included in the analysis. Descriptive characteristics of the sample are presented in Table 1. Mean RWR between the official weigh-in and fight night was 10.17±3.66%. RWR did not differ significantly between organizations. Fighters competing in the UFC demonstrated a mean percent weight change of 10.64±3.28%, whereas Bellator MMA fighters demonstrated a mean percent weight change of 9.80±3.91%. An independent samples t-test indicated that this difference was not statistically significant (p=0.15; Table 2).

**Table 1:** Descriptive characteristics of winning fighters overall and by subgroup Descriptive values are presented as mean±standard deviation (M±SD). UFC: Ultimate Fighting Championship; MMA: mixed martial arts; RWR: rapid weight regain

Variables	All (n=154)	UFC (n=67)	Bellator MMA (n=87)	Men’s divisions (n=129)	Women’s divisions (n=25)
Age (years)	29.88±4.47	29.72±3.82	30.00±4.93	29.62±4.35	31.20±4.94
Height (cm)	175.58±8.37	175.52±9.85	175.63±7.09	177.38±7.38	166.32±7.02
Official weigh-in mass (kg)	151.62±23.36	149.50±25.24	153.25±21.82	156.58±21.84	126.01±10.95
Fight-night mass (kg)	166.82±24.76	165.41±27.66	167.91±22.38	172.39±22.75	138.12±11.13
RWR (%)	10.17±3.66	10.64±3.28	9.80±3.91	10.29±3.67	9.54±3.62

**Table 2:** RWR (%) by organization among winners Independent samples t-test. Statistical significance was set at p≤0.05. UFC: Ultimate Fighting Championship; MMA: mixed martial arts; RWR: rapid weight regain

Organization	n	%	RWR (%) mean ± SD	P-value
UFC	67	43.51%	10.64±3.28	
Bellator MMA	87	56.49%	9.80±3.91	0.147

No significant differences in RWR were observed between sex divisions. Fighters in women’s divisions demonstrated a mean percent weight change of 9.54±3.62%, while fighters in men’s divisions demonstrated a mean percent weight change of 10.29±3.67%. This difference was not statistically significant based on an independent samples t-test (p=0.354; Table 3). Method of victory was not impacted by RWR among winning fighters (Table 4). Fighters who won by KO/TKO demonstrated a mean percent weight change of 10.55±4.50%, compared with 9.30±3.54% for submission victories and 10.18±3.05% for decision victories. A one-way analysis of variance indicated no significant effect of RWR on method of victory (p=0.383).

**Table 3:** RWR (%) by sex division among winners Independent samples t-test. Statistical significance was set at p≤0.05. RWR: rapid weight regain

Division	n	%	RWR (%) mean±SD	P-value
Women’s divisions	25	16.23%	9.54±3.62	
Men’s divisions	129	83.77%	10.29±3.67	0.354

**Table 4:** RWR (%) by method of victory among winners One-way ANOVA. Statistical significance was set at p≤0.05. RWR: rapid weight regain; ANOVA: analysis of variance; KO: knockout; TKO: technical knockout

Method of victory	n	%	RWR (%) mean±SD	P-value
KO/TKO	51	33.12	10.55±4.50	
Submission	24	15.58	9.30±3.54	
Decision	79	51.30	10.18±3.05	0.383

## Discussion

The primary finding of the present study was that RWR did not impact the method of victory among winning professional MMA fighters. Additionally, no significant differences in RWR were observed between organizations or between sex divisions. These findings suggest that while RWR is highly prevalent among successful MMA competitors, the magnitude of weight regained following official weigh-ins does not appear to influence how victories are achieved.

The observed mean RWR of approximately 10% aligns closely with previous investigations in professional MMA athletes. Prior studies in UFC competitors have reported post-weigh-in weight regain ranging from 9%-10%, with similar patterns observed across multiple weight categories [12]. Matthews and Nicholas [10] likewise reported substantial post-weigh-in mass increases in MMA athletes, reinforcing the notion that RWR is a normative practice rather than an exception. The consistency of these findings across organizations and regulatory contexts underscores the entrenched nature of weight-cutting and refeeding strategies within MMA culture.

Despite the widespread belief that greater post-weigh-in weight regain may confer a competitive advantage, the present findings add to a growing body of evidence suggesting that this advantage may be overstated. Previous work in Bellator MMA athletes demonstrated that fighters who regained ≥10% of their body mass were not more likely to win compared to those who regained less weight [21]. Similarly, Schwarz et al. [22] reported no relationship between RWR and competitive success in UFC fighters. The present study extends these findings by demonstrating that, among fighters who do win, the magnitude of RWR does not influence whether victories are achieved via KO, submission, or decision.

The lack of differences in RWR between methods of victory suggests that factors other than acute body mass changes may play a more prominent role in determining how fights are decided. Technical skill, tactical decision-making, cardiovascular conditioning, neuromuscular coordination, and psychological preparedness likely exert a greater influence on competitive outcomes than post-weigh-in body mass alone. Furthermore, although aggressive rehydration and nutritional strategies can restore body mass, prior research indicates that full physiological recovery from RWL may not occur within the available time frame [15,20]. As a result, any theoretical advantages associated with increased body mass may be offset by residual physiological strain.

No significant differences in RWR were observed between organizations or sex divisions, which is consistent with previous research indicating similar weight-cutting and refeeding practices across competitive contexts [13,23]. Although regulatory differences exist between organizations, particularly with respect to hydration testing protocols implemented by ONE Championship, such measures are not currently employed by the UFC or Bellator MMA [16]. Consequently, fighters competing under CSAC-sanctioned events appear to engage in comparable post-weigh-in weight regain behaviors regardless of organization or sex division.

Several limitations should be acknowledged. First, this study relied on publicly available regulatory records, which did not include direct measures of hydration status, body composition, or physiological recovery. Second, although all events were sanctioned by the same athletic commission, weigh-ins occurred across multiple cities and venues, which may introduce minor variability despite standardized procedures. Finally, the observational design precludes causal inference regarding the relationship between RWR and competitive outcomes.

## Conclusions

RWR is a pervasive practice among winning professional MMA fighters; however, the magnitude of weight regained does not appear to influence the method of victory, nor does it differ meaningfully by organization or sex division. These findings contribute to the growing body of literature questioning the competitive value of extreme post-weigh-in weight regain and highlight the need for continued evaluation of weight management practices in MMA from both performance and athlete safety perspectives.

## References

[1] Downey G (2007). Producing pain: techniques and technologies in no-holds-barred fighting. Soc Stud Sci.

[2] Amtmann J, Berry S (2003). Strength and conditioning for reality fighting. Strength Cond J.

[3] McClearen J (2017). “We are all fighters”: the transmedia marketing of difference in the Ultimate Fighting Championship. Int J Commun.

[4] Panja T (2026). UFC’s parent company Endeavor valued at over $11 billion. https://www.nytimes.com/2023/04/03/business/endeavor-tko-wwe-ufc.html.

[5] Maher BS (2010). Understanding and regulating the sport of mixed martial arts. Hastings Commun Entertain Law J.

[6] (2026). Nevada State Athletic Commission: Nevada rules of unarmed combat. https://boxing.nv.gov/uploadedFiles/boxingnvgov/content/faq/sections/2022-09-20-NRUC-OFFICIAL-FINAL%20DRAFT.pdf.

[7] Brito CJ, Roas AFC, Brito ISS, Marins J CB, Córdova C, Franchini E (2012). Methods of body mass reduction by combat sport athletes. Int J Sport Nutr Exerc Metab.

[8] Jetton AM, Lawrence MM, Meucci M, Haines TL, Collier SR, Morris DM, Utter AC (2013). Dehydration and acute weight gain in mixed martial arts fighters before competition. J Strength Cond Res.

[9] Coswig VS, Fukuda DH, Del Vecchio FB (2015). Rapid weight loss elicits harmful biochemical and hormonal responses in mixed martial arts athletes. Int J Sport Nutr Exerc Metab.

[10] Matthews JJ, Nicholas C (2017). Extreme rapid weight loss and rapid weight gain observed in UK mixed martial arts athletes preparing for competition. Int J Sport Nutr Exerc Metab.

[11] Reale R, Slater G, Burke LM (2017). Acute-weight-loss strategies for combat sports and applications to Olympic success. Int J Sports Physiol Perform.

[12] Peacock CA, French D, Sanders GJ, Ricci A, Stull C, Antonio J (2022). Weight loss and competition weight in Ultimate Fighting Championship (UFC) athletes. J Funct Morphol Kinesiol.

[13] Peacock CA, Braun J, Sanders GJ (2023). Weight loss and competition weight comparing male and female mixed martial artists competing in the Ultimate Fighting Championship's (UFC) Flyweight division. Physiologia.

[14] Artioli GG, Gualano B, Franchini E, Scagliusi FB, Takesian M, Fuchs M, Lancha AH Jr (2010). Prevalence, magnitude, and methods of rapid weight loss among judo competitors. Med Sci Sports Exerc.

[15] Franchini E, Brito CJ, Artioli GG (2012). Weight loss in combat sports: physiological, psychological and performance effects. J Int Soc Sports Nutr.

[16] Ricci AA, Evans C, Stull C (2025). International society of sports nutrition position stand: nutrition and weight cut strategies for mixed martial arts and other combat sports. J Int Soc Sports Nutr.

[17] Okamoto B: Chinese MMA fighter (2026). China's Yang Jian Bing dies one day after trying to make weight. https://www.espn.com/mma/story/_/id/14344041/chinese-mma-fighter-yang-jian-bing-dies-trying-make-weight.

[18] Reale R, Slater G, Burke LM (2017). Individualised dietary strategies for Olympic combat sports: acute weight loss, recovery and competition nutrition. Eur J Sport Sci.

[19] Ivy JL (2004). Regulation of muscle glycogen repletion, muscle protein synthesis and repair following exercise. J Sports Sci Med.

[20] Casa DJ, Armstrong LE, Hillman SK (2000). National athletic trainers' association position statement: fluid replacement for athletes. J Athl Train.

[21] Peacock CA, Byers P, Silver T, Antonio J, Sanders GJ, Schwarz A, Stern L (2025). The impact of rapid weight regain on fight outcomes in Bellator mixed martial arts athletes. Cureus.

[22] Schwarz AV, Byers PJ, Stern L, Sanders GJ, Silver T, Antonio J, Peacock C (2025). Rapid weight regain is not linked to success in professional UFC fighters during competition. Internet J Allied Health Sci Pract.

[23] Evans C, Stull C, Sanders G, Ricci A, French D, Antonio J, Peacock CA (2023). Weight cutting in female UFC fighters. J Int Soc Sports Nutr.

